# Transient Population Dynamics of Mosquitoes during Sterile Male Releases: Modelling Mating Behaviour and Perturbations of Life History Parameters

**DOI:** 10.1371/journal.pone.0076228

**Published:** 2013-09-23

**Authors:** Christopher M. Stone

**Affiliations:** 1 Department of Epidemiology and Public Health, Swiss Tropical and Public Health Institute, Basel, Switzerland; 2 University of Basel, Basel, Switzerland; University of Pretoria, South Africa

## Abstract

The release of genetically-modified or sterile male mosquitoes offers a promising form of mosquito-transmitted pathogen control, but the insights derived from our understanding of male mosquito behaviour have not fully been incorporated into the design of such genetic control or sterile-male release methods. The importance of aspects of male life history and mating behaviour for sterile-male release programmes were investigated by projecting a stage-structured matrix model over time. An elasticity analysis of transient dynamics during sterile-male releases was performed to provide insight on which vector control methods are likely to be most synergistic. The results suggest that high mating competitiveness and mortality costs of released males are required before the sterile-release method becomes ineffective. Additionally, if released males suffer a mortality cost, older males should be released due to their increased mating capacity. If released males are of a homogenous size and size-assortative mating occurs in nature, this can lead to an increase in the abundance of large females and reduce the efficacy of the population-suppression effort. At a high level of size-assortative mating, the disease transmission potential of the vector population increases due to male releases, arguing for the release of a heterogeneously-sized male population. The female population was most sensitive to perturbations of density-dependent components of larval mortality and female survivorship and fecundity. These findings suggest source reduction might be a particularly effective complement to mosquito control based on the sterile insect technique (SIT). In order for SIT to realize its potential as a key component of an integrated vector-management strategy to control mosquito-transmitted pathogens, programme design of sterile-male release programmes must account for the ecology, behaviour and life history of mosquitoes. The model used here takes a step in this direction and can easily be modified to investigate additional aspects of mosquito behaviour or species-specific ecology.

## Introduction

One of the most recognized forms of Allee effects that can lead to population extinctions is a lack of successful mating opportunities when population sizes fall below a certain threshold [1]. That reproduction, by essentially creating an artificial Allee effect, rather than induced mortality, could be used as an effective form of pest control was first described by Knipling [2], [3]. He introduced the sterile insect technique whereby the sex ratio of fertile males to females is heavily skewed through the overflooding of an area with sterilized males, potentially leading to local extinction of the pest. The sterile insect technique (SIT) has been applied successfully against various insects of agricultural or medicoveterinary importance, such as the New World screwworm *Cochliomya hominivorax* [4], and tsetse, the vectors of human African trypanosomiasis [5], [6].

Vector control has and continues to play a major role in the global fight against mosquito-transmitted pathogens such as those causing malaria and dengue. The SIT could plausibly become an important component in certain scenarios, for instance where insecticide resistance is prevalent, or for vector species that are not amenable to traditional control measures such as indoor residual spraying, or insecticide-treated bed nets. Trial releases of radiation-sterilized or chemosterilized male mosquitoes have occurred since the late 1950′s with varying degrees of success [7], [8]. With technological advances, such as genetic modification of insects [9], [10] and an improved understanding of mass production of competitive insects, there has been a re-emergence of interest in this area [8].

The SIT for mosquitoes could possibly be most effective as part of an integrated vector management strategy [11], the WHO recommended approach that aims to combine two or more control methods that work in a synergistic manner and are most suited to a particular ecological setting [12]. For instance, SIT might benefit from integration with other control measures that lower the wild population size beforehand, because SIT becomes more effective at lower population sizes, or to be timed for deployment at lows in population recruitment or just prior to seasonal increases in population size [13]. The SIT method can also be used effectively alongside methods that induce mortality in one or more life stages, such as the release of parasitoids or use of insecticides [14], [15]. However, a thorough understanding of the ecology of the target insect is required in order to effectively integrate control measures [16], and little attention has been given so far on how traditional mosquito-control strategies should be implemented alongside SIT. Although the advances in sterile male mosquito releases have led to a recognized need for greater insight into male mosquito biology [17], this has not yet been translated to effective SIT management recommendations that take the mating behaviour and ecology of mosquitoes into account. Here, I explore these issues using simple modifications of a deterministic matrix model. The objective was to see how the following life history and behavioural parameters affect the short-term, transient population dynamics during a simulated sterile-male release programme.

### Polyandry and sperm precedence

While the importance of aspects of mating behaviour, such as polyandry (i.e., a female mating multiple times, either in succession or by remating after a number of gonotrophic cycles have passed). and male mating-competitiveness, have been previously explored using theoretical models [13], [14], [18], [19], less is known about how these factors interact with the degree of last male paternity resulting from sperm precedence or displacement [20]. This interaction is potentially relevant to the amount of suppression that is achieved by an SIT strategy, because when remating occurs, the proportion of a female's fecundity that is due to the last male she mated with will depend on how sperm mixes and whether sperm displacement occurs.

### Male competitiveness and mortality

Differences in multiple male traits can influence how effective released males are at competing with wild type males for wild type females. Examples of such traits are their dispersal ability, mortality, their physiological mating capacity, and ability to compete in a swarm for access to mating opportunities. Additional mortality imposed on sterile males could result from the sterilization process, or from side-effects of colonization (e.g., if mass rearing results in males less capable of responding to floral odours). The importance of mortality costs in conjunction with a reduced competitiveness is investigated. An interaction between mosquito life history traits that could be important to sterile male releases is that both male and female survivorship is usually better described by age-dependent rather than exponential functions [21], [22], [23] and that male mating capability also depends on age and size [24], [25], [26], [27]. While for mass production it may be more convenient to release pupae or newly emerged mosquitoes, if the mating capabilities of males increase over the first week of life it may be more efficient to store the males in a low-mortality lab for 5–6 d before release. The effect of releasing older males rather than 1-d-old males is explored.

### Male harassment

A form of sexual coercion, male harassment, whereby the repeated attempts of males to copulate are costly to females [28] may be of particular relevance to sterile-male releases, because the operational sex ratio becomes heavily skewed over a short timeframe and females potentially encounter males at far greater rates than normal. That male harassment occurs in mosquitoes has been demonstrated in *Anopheles gambiae*: females that were subject to males for only 3 days had a 2-d shorter median lifespan than females that were not, and the authors were able to attribute this to male harassment rather than a different cost of reproduction (egg production, oviposition, etc.) [29]. In *Aedes aegypti*, conclusive evidence of harassment was not found. Although females kept with a high density of males had the lowest survival rate, it was not significantly different from females kept with other females [30]. The effect of including such a mating cost on the effectiveness of sterile male release programmes is addressed.

### Size-assortative mating and consequences for vectorial capacity

An important aspect of mosquito ecology is density dependence operating at the larval stage. Models suggest that through this mechanism, in certain cases, particularly if release rates are insufficient, or in areas adjacent to release sites, population size could increase as a result of the release of sterile males [13], [31], [32], [33]. For genetic methods where mortality does not occur in the embryo but during the pupal stage (as occurs in the release of insects carrying a dominant lethal gene [31]), models predict that this is less relevant, but traditional mosquito SIT strategies may benefit from being supplemented during critical periods with alternative methods of vector control. A simplification underlying previous models is that density dependence affects only larval survivorship, but not the acquisition of reserves and subsequent imago size. This is an oversight because adult size and teneral reserves of mosquitoes affect both adult longevity (under conditions of starvation) and host-seeking behaviour [34], [35], [36], [37], [38], [39], [40], two important components of disease transmission potential. If SIT results in a temporary increase of larger mosquitoes, this raises the question whether it is possible that the vectorial capacity of the population during this time inadvertently is increased (i.e., is a reduction in mosquito abundance offset by an increase in mosquito survivorship and biting rate), and should be considered when disease control, rather than the suppression of a mosquito population, is the aim of a programme.

A potential threat to the success of a sterile release programme is the evolution of a resistant phenotype whereby females preferentially mate with wild-type males. Adult size is important to consider, because one form of preferential mating that may already be present from the outset is size-assortative mating. SIT programmes could be vulnerable to this if they optimize larval rearing methods to produce a standardized, homogenous male population.

### Perturbation analysis of life history traits

To find an effective way to integrate mosquito sterile-male releases into an area-wide integrated vector-management programme, it would be useful to know how perturbations of life history parameters of mosquitoes affect female population size during the course of a sterile male release programme. A perturbation analysis of short-term, transient dynamics [41] could help place sterile male programmes in such a framework: by indicating how changes in parameter values affect the outcome of the model, it may give insight into which life history parameters to target with additional vector control methods throughout different stages of the programme.

The purpose of considering these behavioural complexities was to determine whether they impact the efficacy of SIT programmes, and if so, whether cognizance of them may lead to more effective, integrated methods.

## Materials and Methods

### Model description

**Figure 1:**
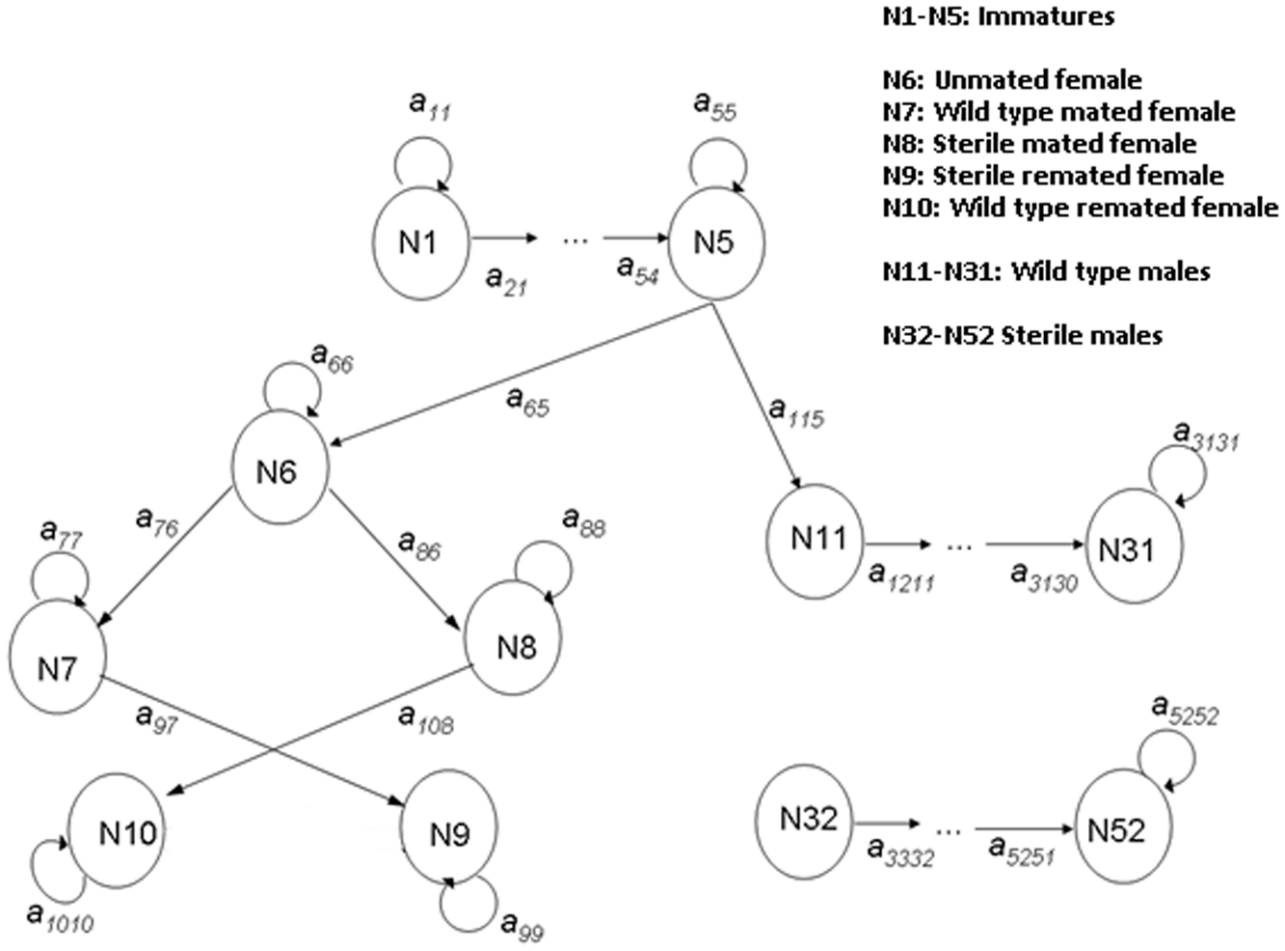
A path diagram of the transitions of the matrix population model employed when larval stages are distinct, to allow for a time lag following oviposition, male mortality is age-dependent, and females mate with wild-type males, sterile males, or both.

Studies on the mating behaviour and mortality patterns of mosquitoes remain relatively scant but have largely focused on, and are divided between two important vectors, the malaria vector *An. gambiae* and dengue vector *Ae. aegypti*. Although the parameter values used for this model come mainly from experiments on *An. gambiae,* certain aspects (e.g., remating, male harassment) may be more relevant to *Aedes* species. Where possible, a comparison between genera is made. The model incorporates density-dependent immature mortality, a mating function, and the release of sterile males. A population projection matrix that encapsulates life stages, female state and male type and age is therefore appropriate [42]. The structure of the base model is depicted in Fig. 1. It is an extension of a stage-structured model used previously to investigate the effects of sugar availability on populations of *Anopheles gambiae* [43]. It consists of immature stages (N1-N5) with density-dependent mortality (see below) and incorporates immature stages to allow for a time delay between oviposition and adult emergence. Female fecundity is assumed to be more strongly influenced by mating status than by age. In a single lifetime, a female can mate with sterile or wild-type males, or both. The latter (indicated in the diagram as “remated”) will be a small portion: first a female has to be receptive to remating (modelled using a “polyandry factor”) and the second mating has to be with a male of a different type, which depends on male ratios. The probability of mating with a wild type or sterile male is given by the following terms: 
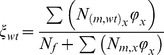


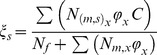



where *φ_x_* is the mating capability of males of age *x* and C is an additional term for competitiveness of released males. In order to keep the model manageable, polyandrous females are assumed to remate only once, where remating indicates an additional copulation leading to insemination. Mated *Anopheles* females are assumed to avoid male swarms, and to remate very rarely [44], while aedine males are assumed to harass or attempt to mate with a female regardless of the female's mating status, suggesting a higher frequency of copulations. Fertility of females mated only with a sterile male is assumed to be zero in the base model. The term 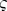 is used to allow the most recent mating to account for a varying proportion of the female's offspring. Males are structured so as to account for age-dependent mortality and age-dependent mating competence. Mating capacity follows Verhoek & Takken [24], with a peak at 7 days. Mortality is based on mesocosm data from Stone *et al*. [23], and a Gompertz-Makeham survival function [45], so that the male age-specific survival probability is
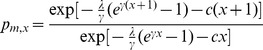



with the following parameter values: λ = 0.0083 and γ = 0.068. An additional mortality for sterile males is included by increasing the value of parameter *c*, as described below. The immature death rate, resulting in an under-compensatory form of density dependence, is given by:
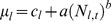



With values as given in Table 1, which were fitted so as to result in a equilibrium female population size of 500–600 mosquitoes. The duration of the immature stage did not depend on larval density and was determined by parameter ε, with a value within the range found in artificial breeding sites [46], [47]. The transition matrix for the model with distinct larval stages and male age-dependent mortality is as follows:
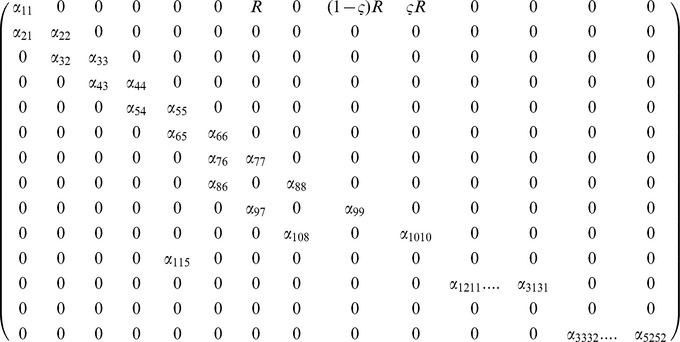



**Table 1:** Rate parameter descriptions and values used.

Parameter	Description	Values used
ε	Eclosion rate	0.14 (immature); 0.7 (per instar)
μ_f_	Female death rate	0.034; 0.028 (large)
λ_gm_	Male mortality factor (Gompertz-Makeham)	0.0083
γ_gm_	Male mortality factor (Gompertz-Makeham)	0.068
c_gm_	Additional male mortality	*varies*
α	Primary sex ratio	0.5
ξ	Mating term	*varies*
γ	Polyandry factor, receptivity to remating	0.02, *or varies*
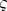	Sperm competition, last male's contribution to offspring	0–1
*c_l_*	Constant immature mortality factor	0.07; 0.2
*a*	Density-dependent immature mortality factor	0.15; 0.2
*b*	Density-dependent mortality modifier	0.15; 0.3
*φ*	Mating capability of males	*varies by age*
*R*	Fecundity/f/d	18
*is*	incomplete sterility of released males	0.03
*C*	Male mating competitiveness	0.1–1

And the transitions between stages are described by the elements of the transition matrix, α_ij_, as follows:
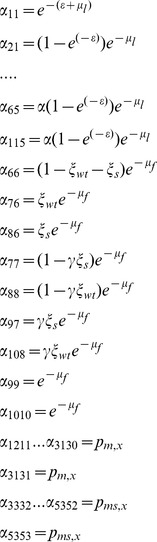



### Simulations

Sterile releases were modelled by assuming 1000 males were released once per week for 20 successive weeks, starting at day 100, at which time the wild type mosquito population was still increasing in size, after being initiated with 100 1^st^ instar larvae only. The effectiveness of the male releases was assessed by comparing the size of the wild type female subpopulation one week after cessation of sterile releases to the female population size in a simulation where the population was projected to the same time point in the absence of sterile releases (referred to as a control). The Matlab code used for these simulations is available from the author upon request.

## Modifications to the model and simulations performed per question

### Polyandry and sperm precedence

To assess the influence of polyandry and sperm precedence both the probability of remating, γ, and the degree of last male paternity,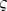, were varied between 0 and 1 by 0.1 increments. For each of these 121 combinations of parameter values a simulation was run as described above and compared to a control simulation where no sterile males were released.

### Male competitiveness and mortality

Mortality of both wild type and released males was assumed to follow a Gompertz-Makeham function, described above, while a constant, *c* (representing additional mortality of released males compared to wild type males), was increased from 0 to 0.25, which encompasses the range of male *An. gambiae* survivorship found in mesocosms with different levels of nectar availability [23]. Competitiveness was varied from 0.1 to 1 by 0.1 increments; cases where competitiveness of released males is greater than 1 (sterile males are hypercompetitive) or 0 (e.g., sterile males form mating aggregations in the wrong sites) were not considered. This resulted in 110 simulation runs that were each compared to the control simulation. A term for incomplete sterility, *is*, of released males was included to explore the importance of this assumption made in the base model, in which case the number of viable offspring produced by females mated with a sterile male becomes the product of daily fecundity and the amount of residual fertility, is*R. Sets of 110 simulations were thus performed with the value of incomplete sterility set at 0% and at 3%.

To assess the impact of releasing older males instead of recently emerged males, depending on the mortality cost of sterile males, the additional mortality parameter, *c*, was increased from 0 to 0.5 in 0.05 increments. A simulation was run for each of these values and compared to the control simulation when releasing 1-d-old males and when releasing 6-d-old males instead.

### Male harassment

Here female survival is considered to be negatively affected by increased male abundance, so that the female death rate is given by:
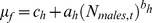



With values of 0.034 (*c_h_*, the base female death rate), and 0.2 (*b_h_*), while the value of *a_h_* is varied between 0 (resulting in the regular death rate) and 0.05 to allow for an investigation of a range of detrimental effects of males on female survivorship. A single simulation run was then performed for each value of *a_h_.*

### Size-assortative mating and consequences for vectorial capacity

An assumption made here is that SIT programmes will typically optimize the number of sterile males produced, rather than the size of males, and that therefore sterile males are of a smaller size than males reared at a low density (but the case where sterile males are larger, or comprise a range of sizes is also explored, see below). To evaluate the effects of size-assortative mating on a suppression strategy, the model is modified into a matrix that keeps track of two groups of mosquitoes: small and larger mosquitoes. The matrix was obtained by simplifying the previous structure to one with only one larval stage and two male stages (teneral males and mature males). The rate at which larvae (N1) become large and small mosquitoes is assumed to depend on larval density:
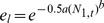



where *a* and *b* represent the same (constant) values used to calculate larval density-dependent mortality. To allow for size-assortative mating, the mating terms are adjusted by including an assortative mating competitiveness term, *C_a_*. For small females, small males are modified by *C_a_*, whereas large males are modified by 1-*C_a_*, and vice versa for large females. Simulations were run with *C_a_* set to 0.5 (i.e., no size-assortative mating) and 0.9 (a high degree of assortative mating) and the impact on female population size tracked. The impact of changes in mosquito biting rates, abundance and survival on the ability of a mosquito population to propagate disease can be investigated by calculating the vectorial capacity of the population [48]:
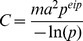



where *m* is the density of mosquitoes (here taken as female population size), *a* the biting rate (0.3 d^−1^ for small females and 0.5 d^−1^ for large females), *p* daily survivorship and *eip* the extrinsic incubation period of the parasite in question (12 d); thus this is a simplified measure of the potential number of infective bites resulting from one infected human. This measure was calculated seperately and summed over large and small female subpopulations.

### Perturbation analysis of life history traits

An elasticity analysis was performed to investigate the effect of small proportional changes in parameter values on female population size [49]. In order to calculate elasticities the projection equation is written as follows:
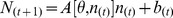



Where θ is a vector of parameters of interest (competitiveness; larval mortality parameters *a*, *b*, and *c*; female and male death rate; incomplete sterility; daily fecundity) and *b_(t)_* a subsidy vector indicating release of sterile males. The base model was simplified to include only one larval stage, two stages for males (teneral and mature), and it is here assumed females only mate once. Instead of weekly pulses of sterile males, the use of a subsidy vector assumes a constant level of release, which was set at 150. The transient sensitivity of n_(t)_ to parameter changes is given by:
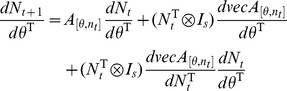



and the elasticity is
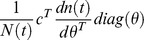



where *c^T^* is a weight vector with zeroes for non-females and ones for females, to calculate elasticity for the female population size [41]. Additionally, because the interest is in perturbations caused by vector control measures, a single death rate *μ_m_* for sterile and wild-type males was used, so that a perturbation in this rate would affect both (because it is unlikely that a control measure could affect males of one type but not the other).

## Results

**Figure 2:**
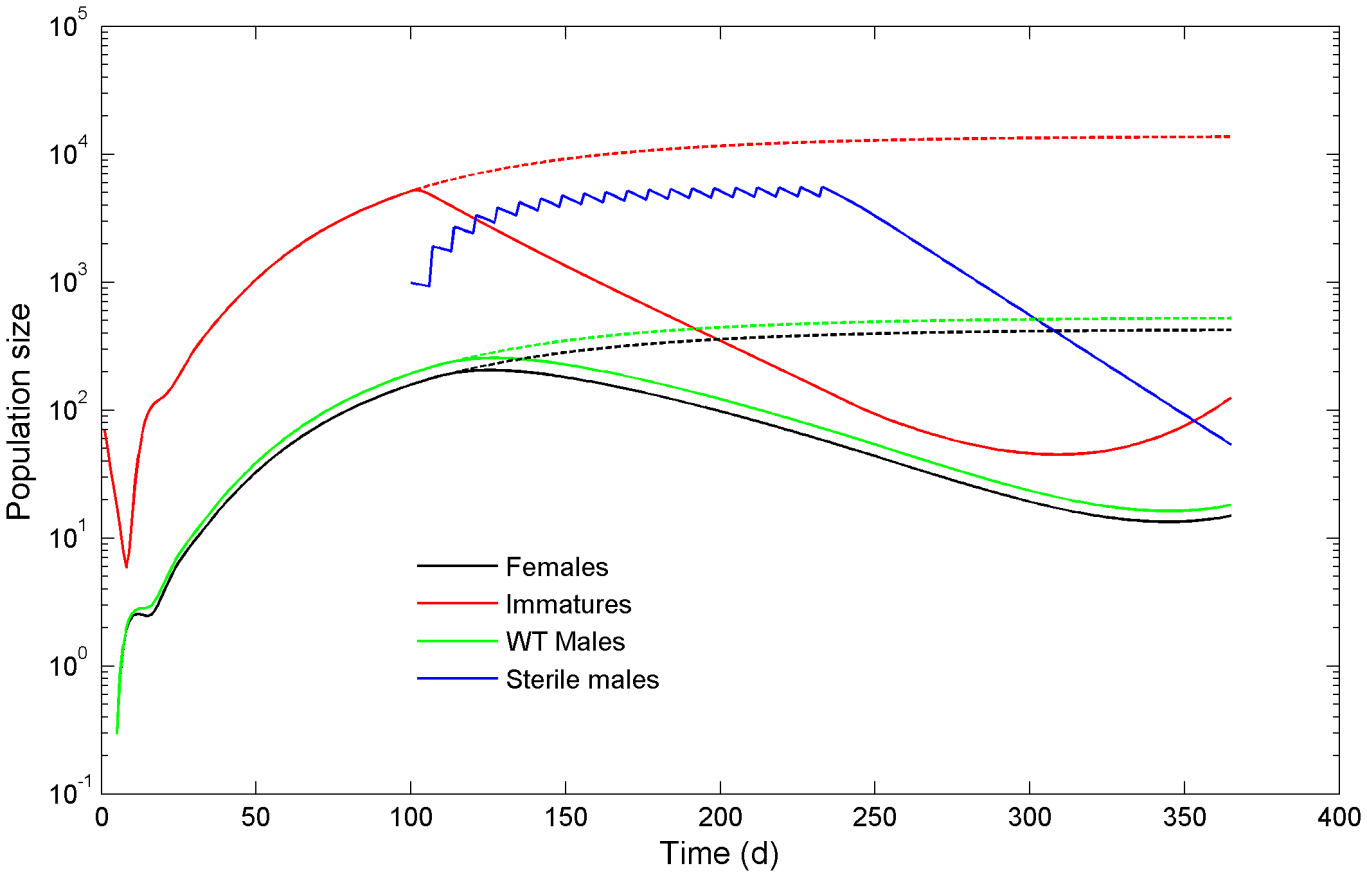
Simulation of a SIT release, showing population sizes of females, wild-type males and larvae, and sterile males that are released at weekly intervals. Dashed lines refer to population sizes in the absence of sterile male releases.

Figure 2 shows the outcome of a straightforward simulation in the presence and absence of sterile male releases, to illustrate how the system functions. The population starts at a very low level (100 immatures) and increases towards, but does not reach, equilibrium over the first 100 days, at which point weekly pulses of 1000 sterile males are released. The number of released sterile males was chosen so that the standing sterile population size during the release period would be approximately ten times the size of the wild type male population. Due to the releases of sterile males there is a dramatic drop in the larval population and a steady decline in adult males and females over the course of 20 weeks. Elimination is not achieved, however, and the population rebounds rapidly at the end of the release period (not shown).

**Figure 3:**
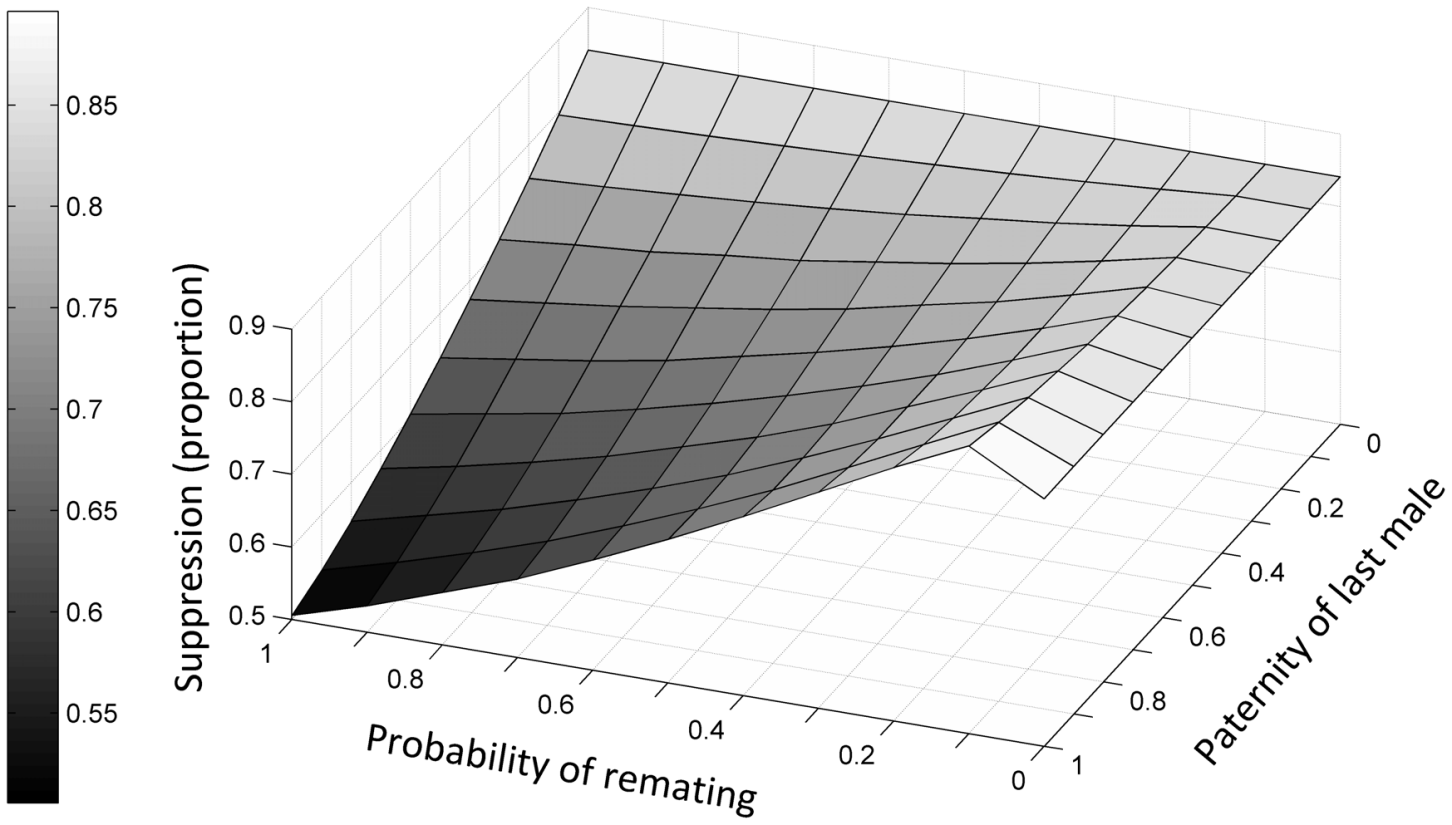
Effects of sperm displacement and probability of remating on female population size after 20 weekly sterile males releases, compared to population size in the absence of sterile males.

Figure 3 explores the effect and interactions between the degree of remating and last male precedence on a suppression strategy, in terms of the degree of suppression achieved one week after cessation of sterile male releases, compared to a control scenario in which no sterile males are released. The effect of an increase in the rate of female remating depends on the paternity attributable to the last mating. Particularly if the last male has precedence, the sterile-male release programme becomes less effective at high rates of remating, but there is a slight increase in the efficiency of the programme at low rates of remating.

**Figure 4:**
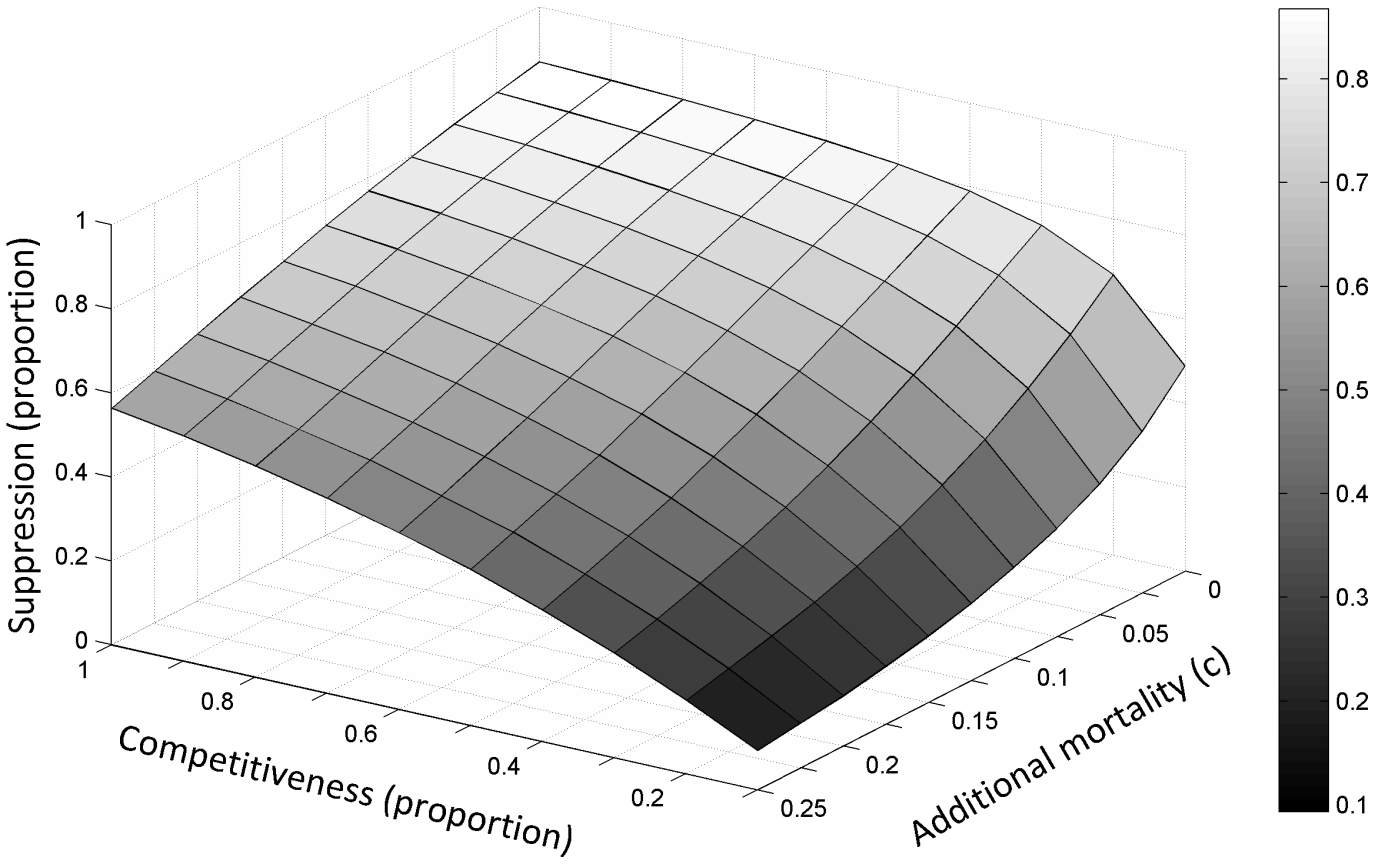
The effect of male mating competitiveness (from 1 to 0.1) and additional mortality incurred by sterile males over wild-type males (expressed as different values of a constant mortality factor in the Gompertz-Makeham survivorship function) on the suppression of the female population achieved after 20 weeks of sterile male releases.

In figure 4 the effect of reduced competitiveness and mortality of released males is explored. Both reductions in competitiveness and mortality make suppression less efficient, but in order for this to be of a magnitude where the female population continues to increase (albeit at a slower rate), rather than decrease, a significant reduction in both competitiveness and mortality is required. This is the case regardless of whether released males are fully sterile or have a small amount (3%) of residual fertility (Fig. S1).

**Figure 5:**
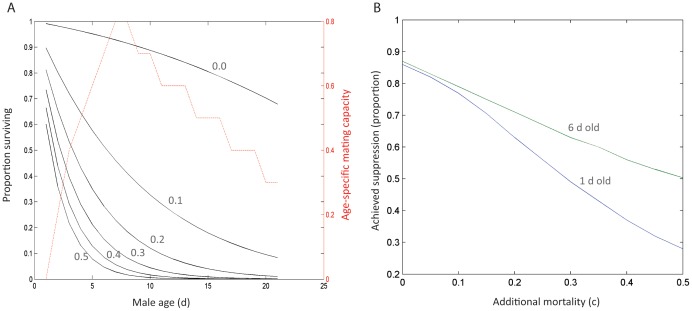
If male mating capacity is age-dependent, the loss of efficacy due to an increased mortality of sterile males can be partially offset by releasing older males, and this effect increases with increasing mortality of sterile males. A) The male age-dependent survivorship and mating capacity curves used in these simulations; B) The difference in the proportion of suppression achieved after 20 weeks of sterile male releases when 6-d-old males are released compared to 1-d-old males.

The implications of releasing older sterile males instead of newly emerged males are depicted in figure 5. Apparent is that when sterile males suffer no or only a small mortality cost over wild-type males there is no appreciable difference in the effectiveness of the sterile release programme, but as the mortality cost of sterile males increases, the advantage of releasing older males increases.

**Figure 6:**
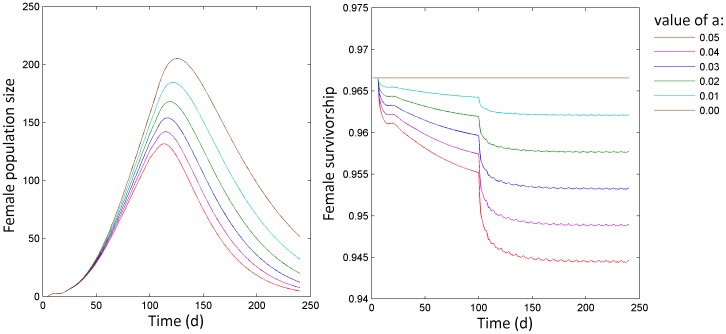
The female population size (left panel) during simulation runs where sterile males are released at weekly intervals starting at day 100 when the female death rate is affected by male harassment (right panel) according to different values of parameter a in the function for male-dependent female mortality.

In figure 6 the relation between female population size during sterile male releases and the value of parameter *a_h_*, which modifies the mortality cost on females associated with male harassment, is depicted. As the effect of males on female mortality increases, the efficacy of the sterile release programme increases: after 20 releases the female population size is 13% of the population without male releases without male harassment (*a_h_* = 0), whereas it is only 2% of the population size without male releases when *a_h_ = *0.05.

**Figure 7:**
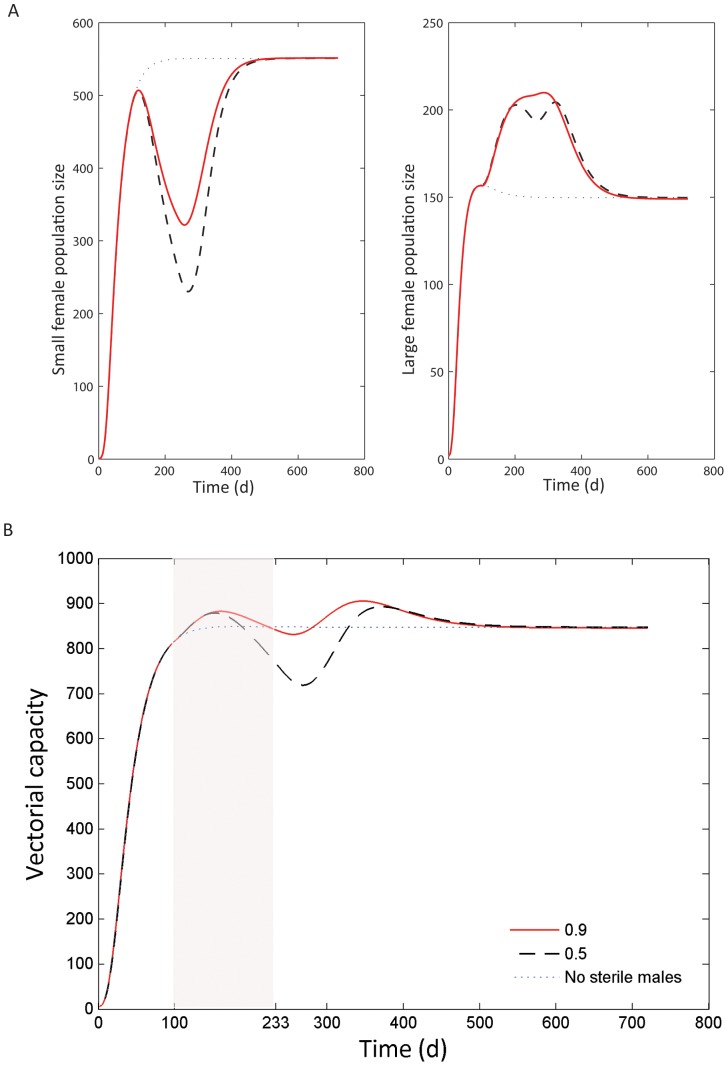
The effect of mosquito size and assortative mating on population size and vectorial capacity during and after the release of sterile males. Solid red lines indicate simulations with a degree of assortative mating, C_a_, equal to 0.9; dashed black lines represent simulations without assortative mating (C_a_ = 0.5). Sterile males were released starting on d 100 for 20 consecutive weeks. Blue dotted lines represent a control where no sterile males were released. A) Population sizes of small (left panel) and large females (right panel). B) Vectorial capacity, a measure of disease transmission potential, of mosquito populations comprising small and large females. The shaded area represents the period during which (small) sterile males are released.

The effect of including size-classes and size-assortative mating into the model is depicted in figure 7a. An interesting point is that suppression strongly reduces the numbers of small females and the larval population. As a result of this, while male releases are ongoing there is an increase in larger females. After the releases of sterile males end, the population returns to its stable state. The greater the degree of assortative mating, the more resilient the population is to suppression. Vectorial capacity throughout the release period is shown in figure 7b. If size-assortative mating occurs (*C_a_* = 0.9), overall vectorial capacity increases during the releases, before returning to the stable state. The overall decrease is more pronounced in the absence of size-assortative mating, but at certain periods during the sterile male releases vectorial capacity increases over the baseline. The result is dependent on the assumption of a higher biting rate of larger females, and a simple countermeasure could be the release of sterile-male mosquitoes of a range of sizes. The latter is confirmed in Fig. S2, which shows the outcomes for female population sizes and vectorial capacity when either large males only or a mixture of large and small males are released.

**Figure 8:**
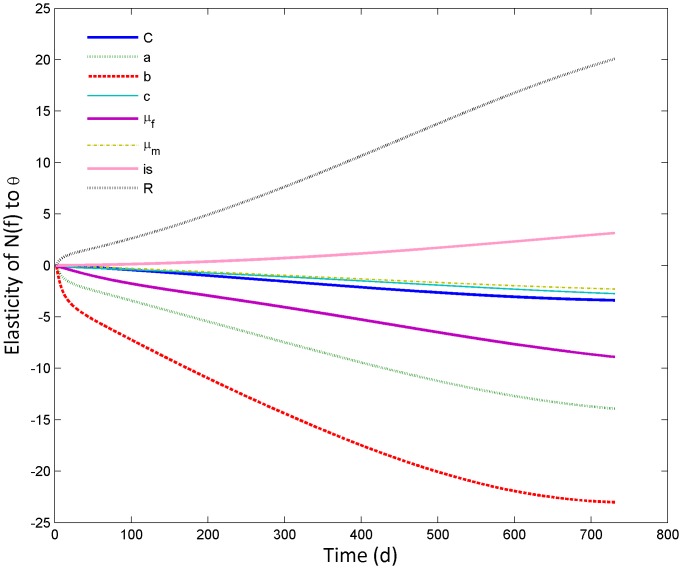
Transient elasticity of female population size to θ, a vector of life history parameters (C, male competitiveness; a,b,c, aspects of larval density dependent mortality; μ_f_, female mortality; μ_m_, male mortality; is, incomplete sterility; R, daily fecundity), throughout the duration of a sterile-male release programme. Release of sterile males was continuous (150 males/day).

Elasticity of female population size to θ, i.e. a vector with the parameters of interest, over the timespan of a sterile male release programme is given in figure 8. Female population size during a sterile male programme is most sensitive to changes in the female death rate, *μ_f_*, female daily fecundity, *R*, and to the immature density-dependent mortality parameters *a* and *b*. Perturbations in components related to male mosquitoes, such as incomplete sterility (*is*), death rate (*μ_m_*) or competitiveness (*C*) are relatively less important.

## Discussion

The objective of this analysis was to bring into focus how aspects of male mosquito life history and mating behaviour influence the outcome of sterile-male release programmes, and how best to integrate this method with other vector control tools. Two components emerge as most deserving of further consideration: the influence of (size-)assortative mating and the observation that decreasing larval competition can undermine the effectiveness of a suppression effort, particularly by resulting in larger mosquitoes with higher biting rates, survivorship, and fecundity. This underscores the importance of developing a better understanding of density dependence acting on immature mosquitoes in terms of development time, survivorship, and body mass. Important questions raised in a recent review [50] are, for instance, how density affects the different larval instars, how it varies seasonally, and how relevant density dependence is in natural breeding sites, shared with other mosquito species and predators. We also need to develop a better understanding of how size and energetic reserves of adult mosquitoes affect their vectorial capacity, and models on vector control methods should take adult size and energetics into account. An example in need of further study that relates to disease transmission is the tendency to take additional (gonotrophically discordant) blood meals under different environmental conditions (for reviews see [51], [52]).

An advantage of the stage-structured matrix model used for these simulations is that state- and age-specific components can be investigated, such as the mating competitiveness, age-specific mating capacity, and age-specific mortality rates of released and wild-type males. A specific recommendation for SIT programmes resulting from this finer definition of male fitness is that if sterile males suffer a significant mortality cost over their wild-type peers, as was recently demonstrated for the genetically modified OX513A strain of *Ae. aegypti* [53], it may be beneficial – from a biological, but not necessarily a cost-effectiveness perspective - to maintain them in a low-mortality laboratory environment for their first week of adult life instead of releasing them immediately after emergence. Such a pre-release period is often employed in fruit fly SIT programmes [54]. This result depends on the assumption that male mating capacity increases over their first week of life, which appears to be the case for both *An. gambiae* and *Ae. aegypti* [24], [25], but perhaps not for all mosquito species. *Aedes albopictus,* for instance, were shown to be fully able to mate once sexual maturation was complete [55], although in another study the mating competitiveness of male *Ae. albopictus* did increase over time, possibly due to an improved nutritional status, leading the authors to likewise suggest keeping males in a laboratory for the first few days before releasing them [56].

The influence of remating and male competitiveness on sterile male releases has been previously investigated using theoretical models. These typically suggested that polyandry would not affect the outcome of such releases if males are equally competitive but would if, for instance, sperm quality or quantity decreases with successive matings [14], [57]. The conclusion from the present analysis agrees with this in general, but not under all circumstances. At low levels of remating, sterile male releases achieved a slightly higher level of suppression than in the absence of remating. Presumably, this occured because the benefits of cancelling out a proportion of the fecundity of previously mated females outweighed the detrimental effects of sterile-male mated females regaining a proportion of their fertility. Whether first or last male precedence occurs in mosquitoes is not entirely clear. In a classic review on sperm competition in insects, a number of studies were cited to suggest that mosquitoes have strong first male precedence [58], but not all these studies made a clear distinction between either copulations or insemination and use of sperm for fertilization. Here, precedence is taken to mean the proportion of offspring fertilized with sperm from the initial versus a second mating. If proportion fertilized is merely due to differences in sperm quantity transferred by males (in this study treated as a part of male competitiveness), neither male has precedence (in this model this is the case if the degree of last male precedence,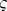, = 0.5). Although female mosquitoes may copulate more than once, typically it is the first mating that counts and instills refractory behaviour in females [59]. When a second copulation does lead to insemination, this is thought to typically occur within a few hours of the first mating before refractoriness has set in, or when the initial mating was disturbed or with a depleted male [60]. Remating has also been linked to females having gone through a number of gonotrophic cycles, presumably depleting the sperm available to them [61]. In the instances where a second copulation does lead to insemination, the progeny tends to be divided between the males, suggesting that if first or last male precedence occurs in mosquitoes, it is not complete [62], [63]. However, most studies reporting polyandry in mosquitoes have provided multiple males at the same time, and are therefore unable to provide insight into precedence. Other studies that have let females mate with one male first and a different male later only report that polyandry has occurred, but not the proportion of offspring due to the first or second mating [59], [64]. Therefore, the question of first or last male precedence remains unresolved for mosquitoes. The analysis reported here suggests that it can impact the effectiveness of SIT programmes, and further studies on this topic may thus be worthwhile. More detailed individual-based theoretical analysis that takes into account, for instance, female refractoriness, quantity of sperm received, and the likelihood of copulations being disturbed by other males (putatively more likely when large numbers of males are released in an area) may also be useful, although it is clear from this study that at the levels at which polyandry occurs in mosquitoes, the impact on SIT programmes will likely be small.

The degree to which male harassment operates in different species of mosquitoes in nature is unknown, but it likely affects mosquitoes in confined laboratory cages to a greater extent and thereby potentially complicates the extrapolation of results from contained, small-scale experiments to a field situation. In nature, for a species such as *An. gambiae*, females locate male swarms when seeking a mate, but otherwise most likely they will not be subject to male harassment. For a species with a host-based mating system, such as *Ae. aegypti*, males may attempt to mate with females while they attempt to blood-feed, but the laboratory evidence is inconclusive. An effect not considered here is that if harassment occurs, females may attempt to avoid this conflict by dispersing to neighbouring areas. This is difficult to study in laboratory settings, but the topic should be investigated further.

The survival values used as baseline in these simulations was based on survival under favourable conditions in a mesocosm, rather than in the field. This choice was made because age-dependent mortality of males under field conditions currently remains unknown and because sterile male fitness costs are most likely be tested under laboratory or semi-field conditions, making comparisons easier. It is likely that under these conditions, male competitiveness and fitness costs affect a suppression effort more than they would using lower rates of survival and resulting higher weekly release rates to maintain a comparable overflooding ratio. Given that even under the favorable conditions simulated these factors were not very influential (for instance, assuming a competitiveness of 0.5, a mortality penalty of *c* = 0.25 –i.e., an additional mortality compared to wild type males, expressed as a constant value in a Gompertz-Makeham function- was required before the suppression effort only stabilized but did not drive the population further down), it seems reasonable to suggest that these components of male fitness are not nearly as critical for further study as behavioural traits (e.g., being able to cue in on appropriate swarm sites, or mating assortatively) that could result in complete mating failure.

Another question facing sterile male programmes is what size male to release. Studies on mating success of different sizes of males of *An. gambiae* suggest that medium-sized males may have an advantage in mating swarms, perhaps due to a combination of energetic reserves and in-flight manoeuvrability [26], [65], although not all studies have confirmed this [27]. To optimize production of sterile males, it may be most efficacious to homogenize the development time and size of the emerging adults [66]. An outcome of this study is that if size-assortative mating already occurs in a mosquito species, as has been shown recently for *An. gambiae* [65], or is something that could easily evolve in the face of prolonged sterile male releases, it may be beneficial to release a mixture of large and small males instead, to avoid potential increases in the mosquito population's vectorial capacity resulting from the reduction in larval density-dependent mortality and development. That this unintended increase as a result of sterile male releases was observed only at high levels of size-assortative mating suggests that other mosquito species, sharing the same larval habitat, could potentially be affected in an even stronger manner. Consequences in terms of abundance of co-existing mosquito species as a result of interspecific competition have previously been modelled [67], and the current results suggest that surveillance of target and non-target mosquito species prior to and during a sterile male programme may also have to take vectorial capacity, rather than merely abundance of vector species, into account.

The inclusion of a large class of mosquitoes illustrates the importance of expressing the impact of control measures in terms of disease transmission potential rather than merely mosquito abundance, as even in the absence of assortative mating, the impact on vectorial capacity is less strong than the reduction in total population size (Fig. 7). Such effects will not be restricted to SIT approaches, but will be most relevant to any vector control method that diminishes mosquito abundance but not the mean age of adult females or the duration of the feeding cycle (e.g., larval control through the use of larvicides). A question that naturally arises is whether combining SIT with another vector control method could help reduce the population and its vectorial capacity during periods where immatures are released from intra-specific competition.

An interpretation of the elasticity analysis is that oviposition site reduction, which would increase or help maintain larval intra-specific competition, would be efficacious. This is indicated by the effect of the density-dependent immature mortality parameter *a*, which Dye [68] describes as being inversely proportional to the number of available larval development sites. It is not obvious how parameter *b* could be perturbed in nature, because it reflects an innate relationship between mortality and population size. It has been pointed out that the value of this factor likely shifts from overcompensating to undercompensating with larval development [68]. Future detailed investigations may therefore want to use instar-specific density-dependence. Methods that target adult females, such as indoor residual spraying or insecticide-treated nets, would likewise be useful, and bed nets may have the additional benefit of increasing the time between successful blood meals and thereby reduce the average daily fecundity of females (*R*). The current insights stem from a perturbation analysis and only point out which life history parameters ideally would be targeted. Further explicit modelling of alternative control strategies alongside SIT should be undertaken to develop this area further, particularly for strategies that may impact two or more life history parameters, such as ITNs, or the implications of, e.g., using larvicides (which would not necessarily maintain high levels of competition) versus oviposition site reduction through environmental management.

The number of investigations of male mosquito life histories and mating behaviour pale in comparison to the number of studies on female behaviour, although progress in this field is being made. For the further development of mosquito SIT it is desirable that these insights are expanded and used to inform and optimize an integrated vector management approach. The results of this analysis apply to a greater or lesser extent to different species, such as *Ae. aegypti* or *An. gambiae*, highlighting the need for species-specific knowledge, particularly on larval density-dependent development, and the effect on resulting female size and disease transmission potential in mosquitoes.

## Supporting Information

Figure S1 The effect of male mating competitiveness (from 1 to 0.1) and additional mortality incurred by sterile males over wild-type males (expressed as different values of a constant mortality factor in the Gompertz-Makeham survivorship function) on the suppression of the female population achieved after 20 weeks of sterile male releases, when released males are not completely sterile (is = 0.03).
(TIF)

Figure S2 **The effect of mosquito size and assortative mating on population size and vectorial capacity during and after the release of sterile males.** Solid lines indicate simulations where 1000 large males are released weekly, dashed lines indicate simulations where a mixture of 500 large and 500 small males are released. The degree of assortative mating, C_a_, is either 0.5 or 0.9. A) Population sizes of small (left panel) and large females (right panel). B) Vectorial capacity, a measure of disease transmission potential, of mosquito populations comprising small and large females. The shaded area represents the period during which sterile males are released.
(TIF)
