# Supplementary figures and images for: Transient Population Dynamics of Mosquitoes during Sterile Male Releases: Modelling Mating Behaviour and Perturbations of Life History Parameters
Source: PLoS One. 2013 Sep 23;8(9):e76228. doi: 10.1371/journal.pone.0076228 (PMC3781073; doi:10.1371/journal.pone.0076228)

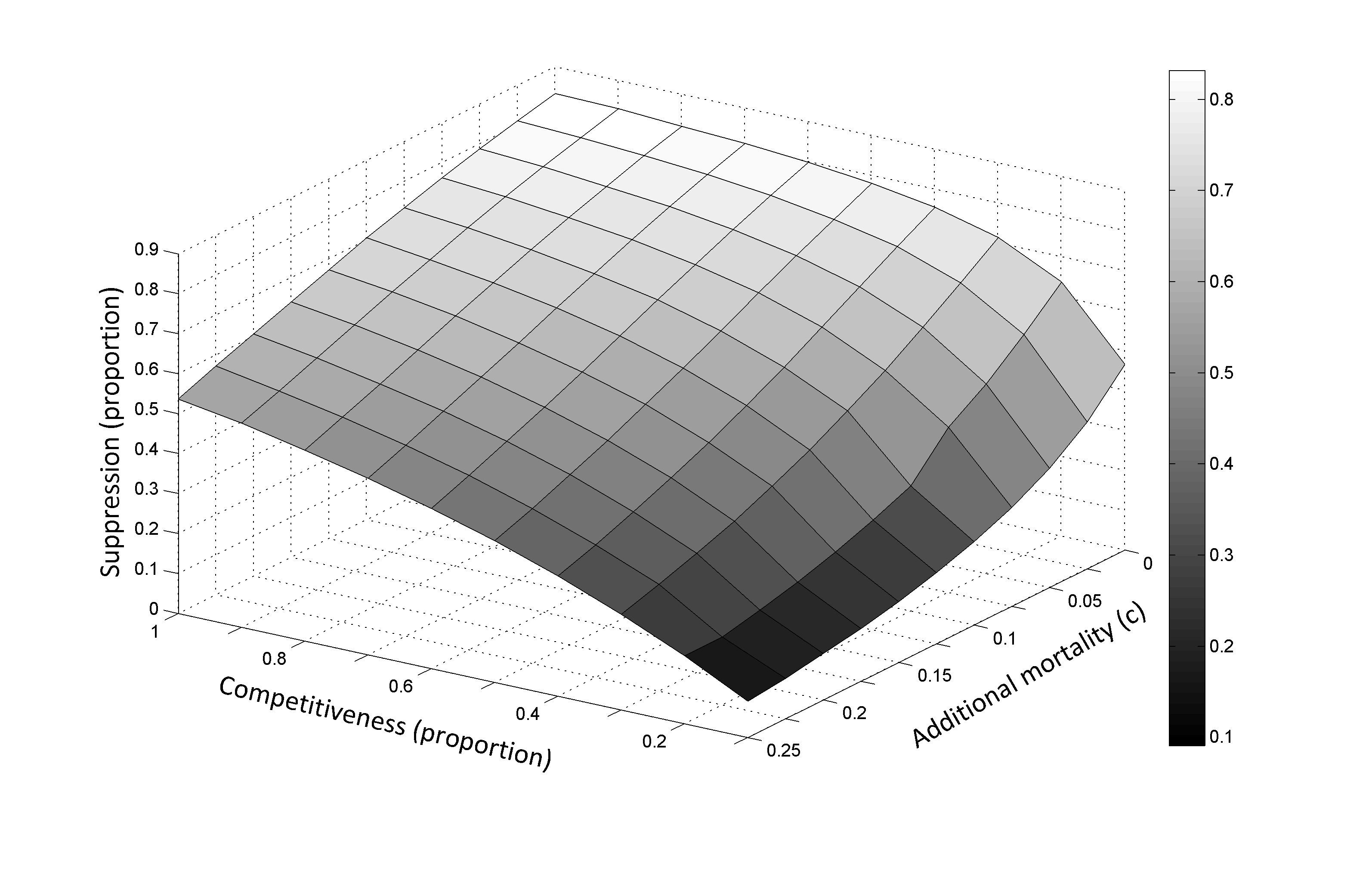

Supplement: Figure S1 — The effect of male mating competitiveness (from 1 to 0.1) and additional mortality incurred by sterile males over wild-type males (expressed as different values of a constant mortality factor in the Gompertz-Makeham survivorship function) on the suppression of the female population achieved after 20 weeks of sterile male releases, when released males are not completely sterile (is = 0.03). (TIF) [file pone.0076228.s001.tif]

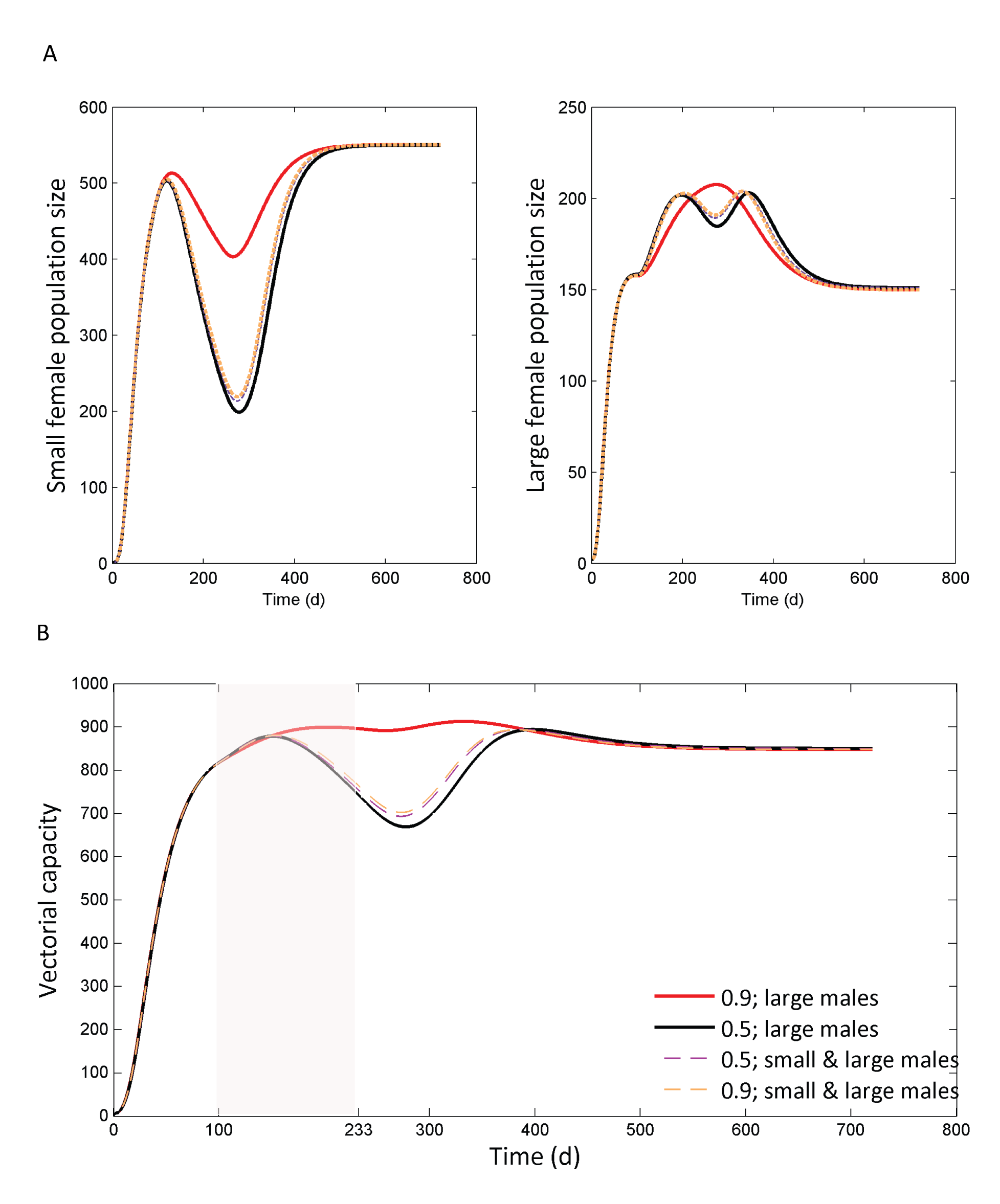

Supplement: Figure S2 — The effect of mosquito size and assortative mating on population size and vectorial capacity during and after the release of sterile males. Solid lines indicate simulations where 1000 large males are released weekly, dashed lines indicate simulations where a mixture of 500 large and 500 small males are released. The degree of assortative mating, Ca, is either 0.5 or 0.9. A) Population sizes of small (left panel) and large females (right panel). B) Vectorial capacity, a measure of disease transmission potential, of mosquito populations comprising small and large females. The shaded area represents the period during which sterile males are released. (TIF) [file pone.0076228.s002.tif]
